# High Prevalence of Tuberculosis and Insufficient Case Detection in Two Communities in the Western Cape, South Africa

**DOI:** 10.1371/journal.pone.0058689

**Published:** 2013-04-01

**Authors:** Mareli Claassens, Cari van Schalkwyk, Leonie den Haan, Sian Floyd, Rory Dunbar, Paul van Helden, Peter Godfrey-Faussett, Helen Ayles, Martien Borgdorff, Donald Enarson, Nulda Beyers

**Affiliations:** 1 Desmond Tutu Tuberculosis Centre, Department of Paediatrics and Child Health, Stellenbosch University, Tygerberg, South Africa; 2 The South African Department of Science and Technology/National Research Foundation Centre of Excellence in Epidemiological Modelling and Analysis, Stellenbosch University, Stellenbosch, South Africa; 3 Department of Clinical Research, London School of Hygiene and Tropical Medicine, London, United Kingdom; 4 National Research Foundation Centre of Excellence for Biomedical Tuberculosis Research, Medical Research Council Centre for Molecular and Cellular Biology, Division of Molecular Biology and Human Genetics, Stellenbosch University, Tygerberg, South Africa; 5 Zambia AIDS Related Tuberculosis Project, University of Zambia Ridgeway Campus, Lusaka, Zambia; 6 International Union Against Tuberculosis and Lung Disease, Paris, France; 7 Department of Clinical Epidemiology, Biostatistics and Bio-informatics, University of Amsterdam, Amsterdam, The Netherlands; The University of Hong Kong, China

## Abstract

**Background:**

In South Africa the estimated incidence of all forms of tuberculosis (TB) for 2008 was 960/100000. It was reported that all South Africans lived in districts with Directly Observed Therapy, Short-course. However, the 2011 WHO report indicated South Africa as the only country in the world where the TB incidence is still rising.

**Aims:**

To report the results of a TB prevalence survey and to determine the speed of TB case detection in the study communities.

**Methods:**

In 2005 a TB prevalence survey was done to inform the sample size calculation for the ZAMSTAR (Zambia South Africa TB and AIDS Reduction) trial. It was a cluster survey with clustering by enumeration area; all households were visited within enumeration areas and informed consent obtained from eligible adults. A questionnaire was completed and a sputum sample collected from each adult. Samples were inoculated on both liquid mycobacterium growth indicator tube (MGIT) and Löwenstein-Jensen media. A follow-up HIV prevalence survey was done in 2007.

**Results:**

In Community A, the adjusted prevalence of culture positive TB was 32/1000 (95%CI 25–41/1000) and of smear positive TB 8/1000 (95%CI 5–13/1000). In Community B, the adjusted prevalence of culture positive TB was 24/1000 (95%CI 17–32/1000) and of smear positive TB 9/1000 (95%CI 6–15/1000). In Community A the patient diagnostic rate was 0.38/person-year while in community B it was 0.30/person-year. In both communities the adjusted HIV prevalence was 25% (19–30%).

**Discussion:**

In both communities a higher TB prevalence than national estimates and a low patient diagnostic rate was calculated, suggesting that cases are not detected at a sufficient rate to interrupt transmission. These findings may contribute to the rising TB incidence in South Africa. The TB epidemic should therefore be addressed rapidly and effectively, especially in the presence of the concurrently high HIV prevalence.

## Introduction

South Africa has high TB and HIV prevalence rates and has not reported a country-wide TB prevalence survey since 1998 [1]. In South Africa the estimated incidence of all forms of TB for 2008 was 960/100000 with an annualised increase in estimated incidence from 1998–2008 of 6.4% [2]. In 2007 it was reported that the total South African population lived in districts with Directly Observed Therapy, Short-course (DOTS) [3] and the National TB programme reported a 64% cure rate amongst new smear positive cases for the same year [4].

In order to determine the TB burden, two TB prevalence surveys have previously been done in communities in the Western Cape Province of South Africa [5]–[6]. At the time of these surveys, the Western Cape had the highest reported rate of all types of TB cases in the country [4]. Den Boon [6] reported a bacteriologically-confirmed TB prevalence of 10/1000 and a smear-positive prevalence of 3/1000 in 2002. Wood [5] reported a point prevalence of adult pulmonary TB cases of 25/1000 with a smear-positive prevalence of 28/1000 in HIV-positive individuals and 2/1000 in HIV-negative individuals in 2005.

This manuscript reports on the prevalence of tuberculosis and the proportion of prevalent cases detected by the TB programme in the Western Cape. In 2005 a survey was done in four communities to inform the sample size calculation and logistical planning for the ZAMSTAR (Zambia South Africa TB and AIDS Reduction) trial, which evaluated the impact of intensified case finding and household contact tracing [7]. Two communities were selected in Zambia [8] and two in the Western Cape, South Africa. The main aim of the analysis for this manuscript was to report on the prevalence of TB in these communities prior to any intervention and we used the indicator ‘patient detection rate’ as a measure of the comprehensiveness of TB services.

## Methodology

### Ethics statement

Approval for the study was obtained from the Health Research Ethics Committees of Stellenbosch University, the University of Zambia and the London School of Hygiene and Tropical Medicine. Participants gave written informed consent for enrolment into the survey and for the collection of sputum and salivary samples. Names of participants were used only on the consent forms. Barcodes were used on questionnaires, sample containers and results and linked data of individuals without using names. Only the data manager could link the barcodes to participants' contact information in order to revisit TB culture-positive participants and refer them to the closest health facility. Participants who were willing to have a salivary HIV test signed a separate informed consent form, received voluntary counselling and testing, and if HIV positive, were referred to the nearest health facility according to the current standard guidelines. Salivary sample results were barcoded and linked to the original sputum samples.

### Study setting

Population-based TB prevalence surveys were carried out in two urban communities in 2005 located in the City of Cape Town. They were purposefully selected for their adjacent location and similarity to the intervention communities of the ZAMSTAR trial as previously described [7]. Each community was served by one TB diagnostic centre defined as a primary health care facility where TB smear microscopy, culture and identification testing were available and patients were diagnosed with and treated for TB.

Community A was a densely populated community with 99% black African residents, of whom 57% lived in shacks and 49% were employed [9]. In 2005 the TB notification rate in this sub-district was 1612/100000 and the antenatal HIV prevalence was 33% [10]. Community B was geographically located in a different sub-district and had a 98% ‘coloured’ population in 2005 of whom 61% lived in brick houses and 77% were employed. In 2005 the TB notification rate in the sub-district was 782/100000 and the antenatal HIV prevalence was 16% [10].

### Study design

Enumeration areas (EAs) from the South African National Census maps of 2001 [9] were randomly selected in each community and placed in order of selection. EAs were included until the target sample size of 5000 participants was reached, but the final EA was completed even if the number went over 5000. Within each EA, all households were visited and households that consented to participate were included in the survey; thus this was a cluster sample survey with clustering by enumeration area. Within each household, all adult household members (aged ≥15 years) who spent the previous night in the household were enumerated and all were eligible for inclusion in the survey. When adults were enumerated but not present at the first visit to their household, two further visits were made to minimize selection bias due to non-response. Trained research assistants obtained written informed consent and interviewed adults using a structured paper-based questionnaire. Variables of interest collected by the questionnaire included age, sex, history of previous TB, number of years resident in the community and socio-economic status assessed by the number of household assets (radio, television, refrigerator, bicycle, car) and the number of meals eaten per day. A sputum sample was requested from each participant. The sample was collected on the spot either spontaneously or with the assistance of breathing techniques.

HIV-prevalence was determined in 2007 on a random subset of the TB prevalence participants. Ethics approval for anonymous HIV testing with BIONOR^TM^ HIV-1&2 was not given prior to the TB prevalence survey in 2005. After completion of the survey, ethics approval was obtained for HIV testing under the condition that a nurse qualified as a HIV-counsellor would test and refer HIV-positive participants to access HIV care. HIV-prevalence was therefore determined 18 months after the completion of the TB-prevalence survey. Salivary samples were taken in 2007 from traced participants.

### Sample size calculation

WHO estimated a TB prevalence of 670/100 000 in South Africa in 2004 [11]. Using the sample size calculated for the primary outcome of the ZAMSTAR trial, which was TB prevalence in the 24 communities included in the trial at the end of the intervention period [7], 5000 adults per community had to be enrolled. However, because of the much higher TB prevalence found while doing the field work, fewer participants were needed to be included in the sample. For the HIV-prevalence at an estimated HIV seroprevalence of 25% and to achieve 5% precision, the calculated sample size was 610 adults from each community.

### Laboratory measurements for TB

Samples were barcoded and transported in cooler boxes to the laboratory. An unstained direct sputum smear was stored for each sputum sample. Samples were then decontaminated and each sample inoculated on both a liquid mycobacterium growth indicator tube (MGIT) (BD, Sparks, USA) and a solid Löwenstein-Jensen (LJ) culture according to standard WHO recommendations. If a MGIT culture was contaminated, the sample was decontaminated again and the culture was repeated using MGIT. Laboratory cross-contamination was investigated as previously described in the Zambian communities [8]. Spoligotyping was done on any sputum samples that had positive cultures that were inoculated on the same day.

The original smear was stained with Ziehl-Neelsen if mycobacteria grew on any culture media. Smears of individuals with a negative culture were not examined. The GenoType® Mycobacterium CM/AS (Hain, Nehren) test was used to identify the species of positive cultures. Laboratory quality was assured for smears by re-reading of the samples by two individuals blinded to the outcome. Drug susceptibility testing was done on culture positive samples with GenoType® MTBDRplus (Hain, Nehren).

### Laboratory measurements for HIV

BIONOR^TM^ HIV-1&2 salivary test kits (BIONOR AS, Norway) were used to collect saliva in the field from participants identified for HIV testing. The collection kits were transported daily to the laboratory where HIV testing was done according to the BIONOR algorithm.

### Case definitions

A culture-positive prevalent pulmonary TB case was defined as an adult who had a positive culture on either medium identified as *M.tuberculosis* species with the GenoType® Mycobacterium CM/AS. A smear positive TB case was defined as an adult with a positive culture identified as *M.tuberculosis (M.tb)* and a direct smear indicating acid fast bacilli. *M.tb* culture-positive participants were revisited within six weeks after the initial visit and were referred for treatment to the local TB clinic after a follow-up questionnaire, the collection of two confirmatory sputum samples for smear and culture and chest x-rays. The same clinical algorithm was used as in the Zambian communities [8].

For study purposes a participant was defined as HIV positive according to the BIONOR algorithm which included a confirmatory test. Participants were counselled and tested voluntarily according to current South African guidelines at the time and referred to the nearest health facility for HIV care if HIV positive.

### Indicator of comprehensiveness of services

The patient diagnostic rate [12] was estimated for each community as the ratio of the notification rate for all pulmonary tuberculosis cases (including retreatment cases) and the age-sex standardised prevalence rates of culture positive tuberculosis cases as estimated from the prevalence survey. Confidence intervals for the patient diagnostic rate were approximated using the Delta method.

### Statistical methods

Data were analysed with STATA 12 (StataCorp LP, College Station, TX, USA). For each community, pulmonary TB point prevalence and 95% confidence intervals were estimated using a logistic regression model with robust standard errors to adjust for clustering at enumeration area level, inverse probability weighting to standardise for age and sex and multiple imputation of missing values on culture-positive TB, as recommended by the WHO [1]. For multiple imputation of missing values, the imputation model included all the variables that were investigated as predictors of culture-positive TB in the multivariable regression model and fifteen imputed datasets were created. Inverse probability weighting was used to account for age-sex differentials in survey participants, and standardisation was to the age-sex structure of the community according to the 2001 census [9]. The design effect for a cluster sample survey, defined as “the multiple by which the sample size must be increased compared to the sample size required if simple random sampling was used, to ensure that the estimate of the population prevalence is as precise as that which would have been obtained from a simple random sample survey” [1], was calculated as the ratio of the variance taking into account the cluster design and the variance ignoring the cluster design [13]. Based on this model, significant predictors of culture-positive TB (p<0.05) in univariable logistic regression were determined. Subsequently, these predictors were included in a multivariable logistic regression model using backwards stepwise selection. The final model consisted of predictors with p<0.05 except for age, sex and previous TB which were included *a priori*. HIV status was not included in the multivariable model because it was measured at a different time point (18 months after the TB prevalence survey) and on a random subset of participants. HIV prevalence was calculated separately for each community to give an indication of background risk.

## Results

### Prevalence

**Figure 1:**
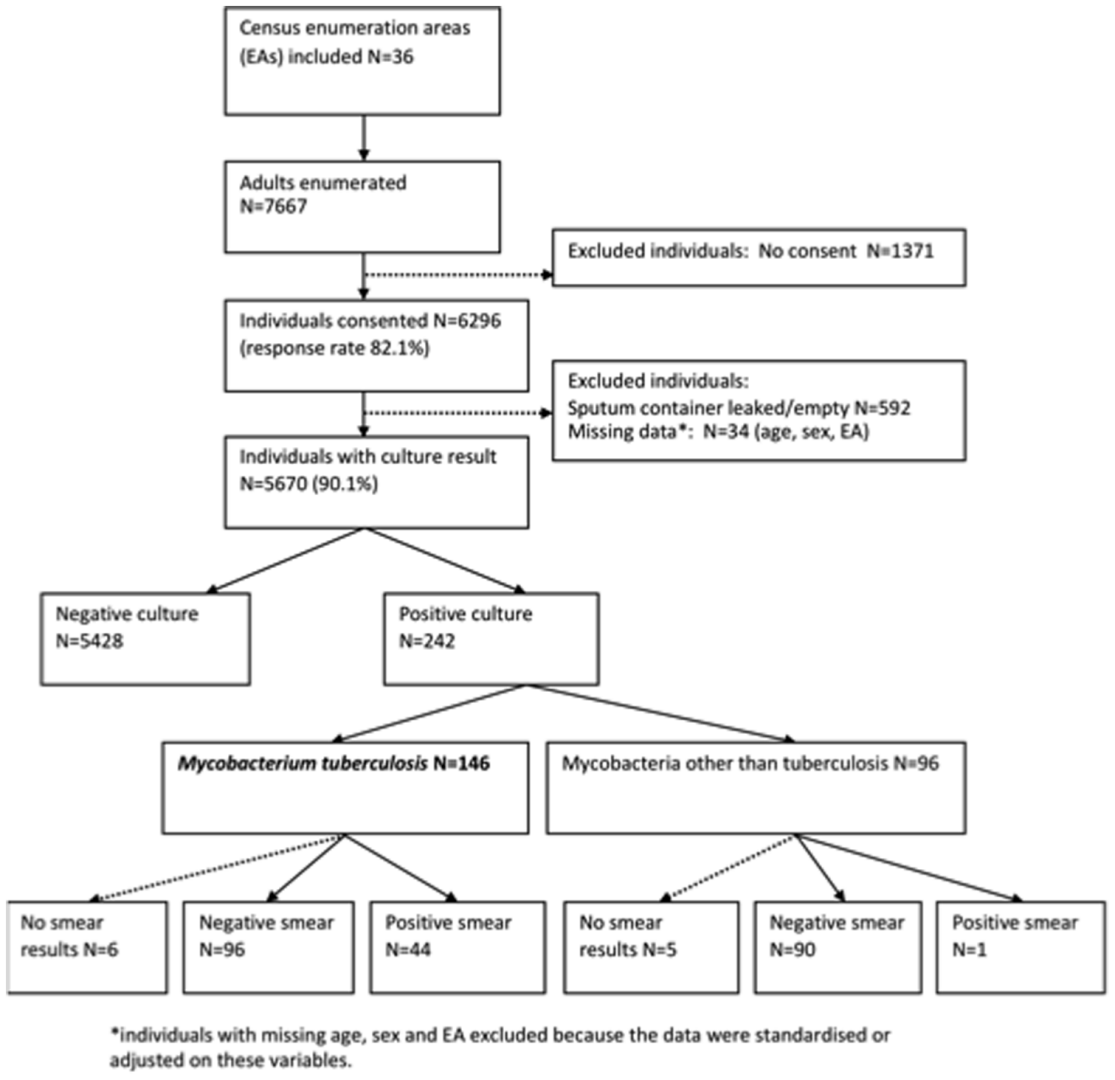
Flow chart of participants included in the survey.

In 36 enumeration areas, 7667 adults were enumerated in the two study communities (**Figure 1**). 6296 adults consented to participate and provided a sputum sample, of whom 5670 adults had culture results and 242 had a positive culture (MGIT and/or LJ). A total of 146 (60%) of the positive cultures were *Mycobacterium tuberculosis (M.tb)*. Forty-four (30%) patients with *M.tb* positive cultures had smears which were direct smear positive. Since patients with any positive culture result (either one of two MGITs or LJ) were defined as culture positive, and because at least one evaluable culture result was available for all participants for whom the sputum sample was not lost, no sample was dropped because of contamination of both cultures. The design effect for Community A was 1.04 and for Community B 2.16.

### Predictors of prevalent TB

Sixty-seven of the culture-confirmed *M.tb* cases were from Community A giving a crude prevalence of culture confirmed TB of 25/1000 (**Table 1**). The crude smear positive prevalence was 7/1000. After standardising for sex and age, accounting for clustering by enumeration area and imputing missing data, the culture prevalence was 32/1000 (95%CI 25–41/1000) and the smear positive prevalence 8/1000 (95%CI 5–13/1000). The increased adjusted prevalence was mainly the result of age/sex standardisation and not of the imputation of missing data. The prevalence was highest in males aged more than 55 years and females 25–34 years. Age and sex were strong predictors of prevalent TB (**Table 2**), as was previous TB (OR = 2.58 95%CI 1.16–5.74), years in community (less TB in participants with longer residence in the area, with overall p = 0.016) and number of household items (less prevalent TB in participants with more household items). Although the number of meals per day was a predictor of prevalent TB in the univariable analysis (OR 0.61 95%CI 0.38–0.98), after controlling for age, sex, previous TB and household assets, there was no longer evidence of an association (OR = 0.99 95%CI 0.49–2.01).

**Table 1:** Prevalence of culture-positive TB in the two communities, overall and by individual and household characteristics.

	COMMUNITY A						COMMUNITY B						
				Cluster, age, sex standardized							Cluster, age, sex standardised		
	N	TB	Prev/1000*	Prev/1000**	95% CI		N	TB	Prev/1000*	Prev/1000**		95% CI	
**Prevalence before imputation**				34	27	43				24		17	33
**Prevalence after imputation**	2726	67	25	32	25	41	3536	79	22	24		17	32
**Sex and age**													
**Male**													
** 15–24**	362	6	17	21	11	42	503	4	8	8		4	18
** 25–34**	233	9	39	46	26	80	299	10	33	34		20	55
** 35–44**	163	11	67	72	37	138	277	10	36	34		18	60
** 45–54**	122	4	33	35	15	80	217	9	41	42		20	88
** 55+**	99	7	71	77	49	120	184	5	27	28		14	53
**Female**													
** 15–24**	603	12	20	23	14	36	599	13	22	22		13	36
** 25–34**	448	9	20	24	14	42	396	10	25	26		13	51
** 35–44**	334	5	15	19	8	44	432	12	28	29		16	53
** 45–54**	185	3	16	19	6	57	360	3	8	9		3	26
** 55+**	177	1	6	6	1	48	269	3	11	11		3	34
**ever TB**													
** no**	2308	46	20	26	19	34	2992	56	19	20		14	28
** yes**	404	21	52	69	38	123	538	23	43	45		25	78
** missing**	14	0					6	0					
**how many meals**													
** less than 3**	1243	39	31	40	30	53	1264	29	23	24		14	41
** 3 or more**	1349	26	19	25	17	36	2105	46	22	24		16	35
** missing**	134	2					167	4					
**years in community**													
** 0–4**	786	25	32	46	32	65	365	7	19	19		11	32
** 5–9**	455	12	26	31	18	55	282	4	14	15		6	37
** 10–14**	378	11	29	33	18	61	262	5	19	23		9	54
** 15–19**	482	9	19	20	11	37	700	17	24	25		16	40
** 20–24**	609	10	16	23	13	39	601	11	18	18		11	30
** 25+**							1299	34	26	29		18	45
** missing**	16	0					27	1					
**house hold items**													
** 0**	353	13	37	51	29	90	136	5	37	45		18	111
** 1**	392	12	31	43	17	105	163	9	55	48		27	85
** 2**	467	15	32	43	29	63	386	18	47	50		32	77
** 3**	1077	23	21	26	16	42	1679	35	21	22		16	31
** >4**	434	4	9	10	4	25	1172	12	10	13		7	24
** missing**	3	0											

* without imputation of missing data.

** with imputation of missing data.

**Table 2:** Univariable and multivariable analysis of predictors of culture-positive prevalent TB in the two communities.

	COMMUNITY A						COMMUNITY B					
	UNIVARIABLE			MULTIVARIABLE			UNIVARIABLE			MULTIVARIABLE		
	OR	95% CI		OR	95% CI		OR	95% CI		OR	95% CI	
**Sex and age**		p<	0.001		p<	0.001		p =	0.012		p =	0.039
**male**												
** 15-24**	1.00			1.00			1.00			1.00		
** 25-34**	2.21	1.02	4.79	1.84	0.82	4.13	4.07	1.86	8.93	3.59	1.52	8.46
** 35-44**	3.55	1.32	9.57	2.60	1.10	6.14	4.07	1.96	8.45	3.37	1.50	7.53
** 45-54**	1.65	0.56	4.86	1.27	0.40	4.06	5.15	1.93	13.70	4.76	1.66	13.65
** 55+**	3.81	1.62	8.96	3.58	1.39	9.19	3.34	1.23	9.07	2.72	0.95	7.81
**female**												
** 15-24**	1.00			1.00			1.00			1.00		
** 25-34**	1.06	0.51	2.21	0.96	0.47	1.96	1.18	0.60	2.31	1.09	0.56	2.15
** 35-44**	0.82	0.29	2.33	0.81	0.27	2.41	1.36	0.56	3.31	1.18	0.48	2.95
** 45-54**	0.82	0.27	2.49	0.98	0.27	3.60	0.40	0.12	1.30	0.38	0.11	1.30
** 55+**	0.27	0.03	2.35	0.31	0.03	2.88	0.49	0.15	1.58	0.47	0.15	1.44
**ever TB**		p =	0.009		p =	0.020		p =	0.011		p =	0.116
** no**	1.00			1.00			1.00			1.00		
** yes**	2.83	1.29	6.19	2.58	1.16	5.74	2.34	1.22	4.50	1.84	0.86	3.91
**how many meals**		p =	0.041					p =	0.988			
** less than 3**	1.00						1.00					
** 3 or more**	0.61	0.38	0.98				0.99	0.49	2.01			
**years in community**		p =	0.012		p =	0.016		p =	0.258			
** 0-4**	1.00			1.00			1.00					
** 5-9**	0.67	0.36	1.25	0.69	0.38	1.24	0.81	0.25	2.57			
** 10-14**	0.72	0.36	1.44	0.74	0.35	1.54	1.20	0.38	3.76			
** 15-19**	0.43	0.23	0.78	0.45	0.24	0.85	1.34	0.80	2.25			
** 20-24**	0.49	0.23	1.02	0.52	0.21	1.34	0.96	0.39	2.37			
** 25+**							1.52	0.75	3.09			
**household items**		p =	0.002		p =	0.013		p<	0.001		p<	0.001
** 0**												
** 1**												
** 2**												
** 3**												
** >4**	0.74	0.61	0.89	0.78	0.64	0.95	0.67	0.60	0.76	0.70	0.61	0.80

Seventy-nine cases were from Community B giving a crude prevalence of culture confirmed TB of 22/1000. The crude smear positive prevalence was 7/1000. After standardising for sex and age, accounting for clustering by enumeration area, and imputing missing data, the culture prevalence was 24/1000 (95%CI 17–32/1000) and the smear positive prevalence 9/1000 (95%CI 6–15/1000). The prevalence was highest in males aged 45–54 years and females aged 35–44 years (**Table 2**). Age and sex were strong predictors of prevalent TB, as was number of household items (less prevalent TB in participants with more household items). 81% of the community had ≥3 household items in comparison with 55% in Community A. There was no evidence of an association between number of meals per day and prevalent TB, in either univariable or multivariable analysis.

### HIV background risk

In the sub-study investigating HIV status, 468 individuals were enrolled in Community A, of whom 17 were TB cases as defined in the TB prevalence survey and 109 were HIV positive. The age, sex and cluster adjusted HIV prevalence was 25% (95%CI 19–30%) among non-TB cases. In Community B, 672 individuals were enrolled of whom 36 were TB cases and 166 were HIV positive. The age, sex and cluster adjusted HIV-prevalence was 25% (95%CI 19–30%) among non-TB cases.

### Patient diagnostic rate

In Community A the patient diagnostic rate was 0.38 per person-year (**Table 3**). The patient diagnostic rate was 0.9 per person-year among smear positive and 0.11 per person-year among smear-negative patients. In Community B the patient diagnostic rate was 0.30 per person-year. The patient diagnostic rate was 0.59 per person-year among smear positive and 0.04 per person-year among smear-negative patients.

**Table 3:** Estimates of patient diagnostic rates for pulmonary tuberculosis.

Population	(a) Notification rate of PTB/1000 Population	(b) Prevalence of PTB/1000 Population	(a)/(b) Patient Diagnostic Rate*	95% Confidence Interval
**Community A**				
Smear Positive	7.73	8.60	0.90	0.47–1.33
Smear Negative	2.12	20.22	0.11	0.08–0.13
Total	12.31	32.79	0.38	0.29–0.47
**Community B**				
Smear Positive	4.57	7.75	0.59	0.38–0.90
Smear Negative	1.37	37.95	0.04	0.03–0.05
Total	7.00	23.58	0.30	0.20–0.39

* Expressed as the number of cases detected per person-year among persons with PTB.

### Spoligotyping of M.tb strains

Spoligotyping showed that samples collected and prepared for culture on the same day were similar in 12 samples on 6 separate days (2 per day). Six of these samples were Beijing strain, four were F11 strain and two Haarlem strain. Although the possibility exists that these samples may have been cross contaminated giving a contamination proportion of 6 out of 242 positive cultures (2.5%), the laboratory methodology used (spoligotyping) could not differentiate between substrains of Beijing, F11 or Haarlem. It is therefore also possible that these samples were not contaminated but contained substrains commonly found in the South African context. In addition, given that these are the most dominant strains found in our communities, the probability also exists that these were correctly identified and not contaminants.

### Drug susceptibility testing

Of the 146 prevalent TB cases, 141 had drug susceptibility test results and of these, six cases were isoniazid mono-resistant (4%), one case was rifampicin mono-resistant (1%) and one case was isoniazid and rifampicin resistant (1%). The rest of the samples were sensitive to isoniazid and rifampicin.

## Discussion

This paper reports a very high prevalence of culture positive pulmonary TB in the communities studied. The patient diagnostic rate was much higher for smear positive patients than for smear negative patients, amongst whom it was very low.

The estimated TB prevalence according to the WHO [14] for South Africa was 5/1000 in 2005. The prevalence of smear positive and culture positive TB reported in both communities were higher than the national estimates which could be explained by the fact that high burden TB communities were purposefully selected in accordance with the ZAMSTAR protocol. Nevertheless, this extremely high TB prevalence, which was measured with high quality survey methods, is cause for concern, in particular when taking into account the low patient diagnostic rate, suggesting the services fail to detect a significant proportion of TB cases. Moreover, the notification rates for both subdistricts showed a steady increase in TB incidence [10], [15], suggesting that current services are not stemming the tide of TB in these communities.

Given the limited geographical coverage of surveys in South Africa, significant effort should be made to improve prevalent TB data estimation in order to inform policymakers and implementers of the TB programme, hence the planning of a national TB prevalence survey by the National TB Programme of South Africa. It is crucial to address the TB epidemic in South Africa immediately and effectively since the latest WHO report [16] indicated South Africa as the only country where TB incidence is rising. The fact that the incidence is rising and the prevalence is so high suggests an epidemic still on the upwards curve.

In Community A, the informal settlement with a dynamic population, there was less TB in participants with longer residence in the area. This may be a proxy indication of the influence of intraregional migration on prevalent TB. However, migrants into community A are mostly from the Eastern Cape [10] which has a reported lower TB notification rate than the Western Cape. The migration stream from the Transkei region in the Eastern Cape has been the key demographic flow into the Western Cape and has transferred large numbers of individuals and families from rural areas. This migration stream deposits poor individuals and families into the informal urban settlements surrounding Cape Town. Given the known association between poverty and TB [17], this group may be at high risk of developing TB.

In both communities the study estimated a low patient diagnostic rate, suggesting that cases are not detected at a sufficient rate to interrupt transmission. A patient diagnostic rate of >1.17 indicates more than 70% of cases are detected as proposed [18] as part of an effective DOTS strategy. However, the heterogeneity between the two communities was also demonstrated by the higher patient diagnostic rate among smear-positive patients in Community A (0.99 per person-year) compared to Community B (0.50 per person-year). For Community A this is an indication of a well functioning TB programme, although the detection of smear-negative TB is still not ideal. Solutions could be tailored to address this epidemic heterogeneity.

Communities included in this study thus had a higher TB prevalence and poorer case detection than national estimates [4]. The introduction of GeneXpert (Cepheid, USA) which is currently been rolled-out across South Africa may improve case finding. While limited case detection may be an important contributor to the high TB prevalence and may contribute to the rising incidence of TB in South Africa, other factors may play a role as well, such as inadequate treatment success (reported to be 70% in the 2007 WHO report [14] on the 2004 treatment cohort for the same time period as this study).

Different risk groups were identified during the survey in the two communities, specifically males of all ages, those having had previous TB (as was found in a similar South African community [6]) and those of lower socio-economic status (as indicated by number of household items). While this confirms epidemiological expectations, our findings do not identify clearly defined risk groups for intensified case finding. Other Southern African surveys [8], [19] indicated previous TB as a risk factor only in HIV positive individuals. The final outcome prevalence survey of the ZAMSTAR trial will however inform on the proportion of prevalent TB that is attributable to HIV infection in Western Cape since HIV status was captured on an individual level.

With regards to the HIV prevalence in the communities, it was expected that the prevalence survey would mirror the antenatal care HIV prevalence as was reported by the provincial health system. The antenatal HIV prevalence in the subdistrict of Community A was 16% and for the subdistrict of Community B it was 33% in 2005 [10]. However, according to the survey, HIV prevalence was similar across the two communities: lower in Community A, but higher than expected in Community B with both communities' survey HIV prevalence at 25%. This may be an indication of subsets of HIV epidemics within the province, with a HIV prevalence on the rise in some communities, but already plateauing in other communities. An upwards trend in HIV prevalence was illustrated [20] in both subdistricts from 2001 to 2004 with an increase in prevalence from 22% to 33% in the Community A subdistrict and an increase of 5% to 15% in the Community B subdistrict^,^ thus also illustrating the heterogeneity of the HIV epidemic within the Western Cape province, and indicating significant increases in the HIV prevalence in both the study subdistricts just prior to our study. This will undoubtedly further exacerbate the already perilous situation of tuberculosis in these communities.

### Limitations

The most important limitation of the survey was that we could not directly investigate HIV status as a predictor for prevalent TB. However, the association between HIV and TB is well established. As was recently recommended by the WHO [1], testing for HIV should at least be included for all participants confirmed to have TB and may be considered for a wider group of survey participants in TB prevalence surveys, especially in settings where the HIV prevalence is high in the general population and where HIV testing is a routine service. It is strongly recommended that the South African National TB Programme includes testing for HIV offered to all participants as part of the planned national TB prevalence survey.
